# The complete chloroplast genome of *Epimedium campanulatum* Ogisu (Berberidaceae), a rare plant species endemic to China

**DOI:** 10.1080/23802359.2021.2024771

**Published:** 2022-03-13

**Authors:** Yixin Zhang, Xiang Liu, Cheng Zhang, Chaoqun Xu, Weihan Qin, Guoan Shen, Baolin Guo

**Affiliations:** a Institute of Medicinal Plant Development, Chinese Academy of Medical Science, Peking Union Medical College, Beijing, China; b Chongqing Academy of Chinese Materia Medica, Chongqing, China; c Key Laboratory of Biodiversity Science and Ecological Engineering, Ministry of Education, College of Life Sciences, Beijing Normal University, Beijing, China

**Keywords:** Chloroplast genome, *Epimedium campanulatum*, infrageneric classification, phylogenetic analysis, Berberidaceae

## Abstract

*Epimedium* L. is an important medicinal herbaceous genus that belongs to the family Berberidaceae. *Epimedium campanulatum* Ogisu is a plant species only inhabited in the northwestern part of Sichuan province, China. Here, we reported the complete chloroplast genome sequence, assembly, and characterization of *E. campanulatum*. The chloroplast genome of *E. campanulatum* was 157,343 bp in length, and a total of 112 unique genes were identified. Phylogenetic results revealed that *E. campanulatum* formed a sister relationship with the cluster of *Epimedium ecalcaratum*, *Epimedium davidii*, and *Epimedium chlorandrum*. Our findings provided valuable data for future taxonomic and phylogenetic research within the genus *Epimedium*.

*Epimedium* L., the largest herbaceous genus within the family Berberidaceae, contains more than 60 species (Stearn 2002; Ying 2002). Due to their special therapeutic effects on kidney, bones, and muscles, the leaves of *Epimedium* plants have long been used as an important herb "Herba Epimedii" in Traditional Chinese Medicine (Ma et al. 2011; Fan and Quan 2012; Yang et al. 2019). However, the infrageneric classification of *Epimedium* genus has remained controversial all along (De Smet et al. 2012). Chloroplast genomes are regarded as an important tool used in phylogenetic research due to their special advantages (Nock et al. 2011; Zhang and Li 2011; Li et al. 2015). In this study, we reported the first complete chloroplast genome of *E*. *campanulatum*, which is a morphologically unique species that was narrowly distributed in the northwestern part of Sichuan province, China (Ogisu 1996).

The *E*. *campanulatum* sample was collected from Longchi County, Dujiangyan city of Sichuan province, China (latitude 31.1068 and longitude 103.5571). The specimen and extracted DNA were deposited at Medicinal Plants Authentication Center, Institute of Medicinal Plant Development, Chinese Academy of Medical Science (http://www.implad.ac.cn/, contact Baolin Guo, blguo@implad.ac.cn) under the voucher number C. Zhang274. The genomic DNA was extracted from the fresh leaves of *E*. *campanulatum* with the modified CTAB method (Doyle and Doyle 1987), and was then used to generate libraries with an average insert size of 300 bp using the VAHTSTM Universal DNA Library Pren Kit (ExCell Bio. Biological Technology Co., Ltd., Shanghai, China). Genome sequencing was conducted with the Illumina Novaseq 6000 platform (Illumina Inc., San Diego, CA), and 150 bp paired-end reads were generated. The assembly of chloroplast genome was performed using the GetOrganelle v1.5 program (Jin et al. 2018) with *E. acuminatum* (GenBank accession number: NC_029941) as reference. The annotation of chloroplast genome was conducted through the online program CPGAVAS2 (Shi et al. 2019) and assisted with manual correction, and the annotated genomic sequence was deposited into GenBank with an accession number (MW470954).

The complete chloroplast genome of *E*. *campanulatum* (MW470954) was 157,343 bp in length, including two inverted repeat regions (IR_A_ and IR_B_, 26,045 bp) separated by a large single-copy region (LSC, 88,175 bp) and a small single-copy region (SSC, 17,078 bp). The total GC content was 38.79%, and the GC content of IR regions (43.16%) was higher than that in LSC (37.37%) and SSC regions (32.73%). A total of 112 unique genes were identified from the chloroplast genome of *E*. *campanulatum*, including 78 protein-coding genes, 30 tRNA genes, and four rRNA genes. The intron-exon structure analysis indicated that a total of 18 genes have introns, among which 15 genes contain one intron and three genes contain two introns.

The phylogenetic analysis was conducted using the complete chloroplast genome sequences of *E*. *campanulatum* and other 14 species downloaded from the NCBI GenBank database. MAFFT v7 (Katoh et al. 2019) was used to generate sequence alignment. The maximum-likelihood (ML) analysis was conducted using the RaxmlGUI v1.5b2 program (Silvestro and Michalak 2012) and the Bayesian inference (BI) analysis was conducted using MrBayes 3.2.7 (Ronquist and Huelsenbeck 2003). *Vancouveria hexandra* was selected as the outgroup (Figure 1). As a result, the ML and BI phylogenetic tree displayed identical topologies, demonstrating that *E. campanulatum* was sister to the cluster of *Epimedium ecalcaratum*, *Epimedium davidii*, and *Epimedium chlorandrum*. Our study provided valuable information for facilitating future phylogenetic and evolutionary studies of *Epimedium* genus.

**Figure 1.**
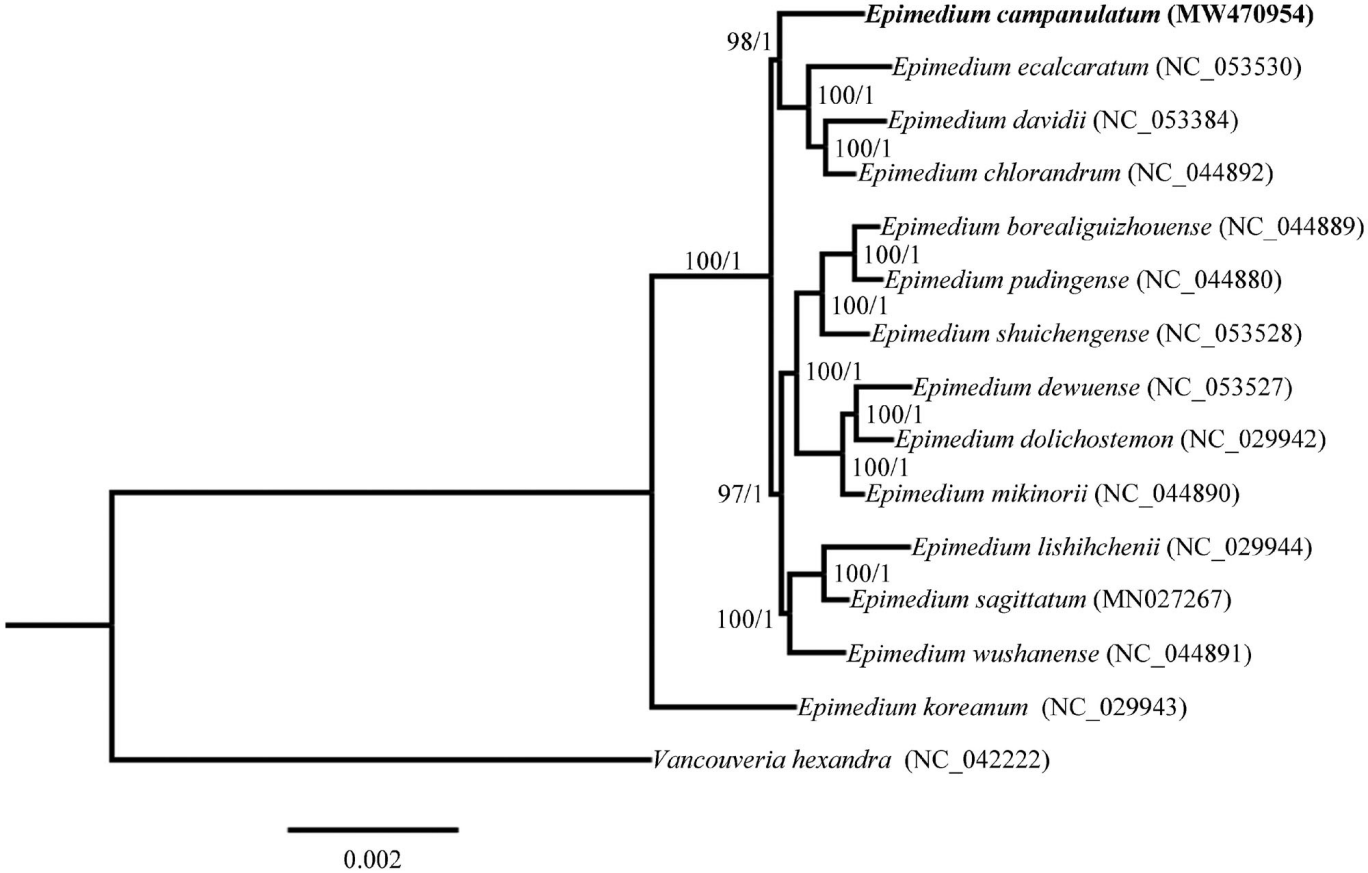
Maximum-likelihood (ML) and Bayesian’s inference (BI) phylogenetic tree based on the complete chloroplast genomes of 15 species, with *Vancouveria hexandra* as outgroup. The support values at the nodes represent maximum-likelihood bootstrap support (1000 replicates) and Bayesian’s inference posterior probabilities.

## Data Availability

The genome sequence data that support the findings of this study are openly available in GenBank of NCBI at https://www.ncbi.nlm.nih.gov/ under the accession no. MW470954. The associated numbers are PRJNA763305, SRR15926849, and SAMN21437724.
